# Among three different executive functions, general executive control ability is a key predictor of decision making under objective risk

**DOI:** 10.3389/fpsyg.2014.01386

**Published:** 2014-12-03

**Authors:** Johannes Schiebener, Elisa Wegmann, Bettina Gathmann, Christian Laier, Mirko Pawlikowski, Matthias Brand

**Affiliations:** ^1^General Psychology: Cognition, University of Duisburg-EssenDuisburg, Germany; ^2^Erwin L. Hahn Institute for Magnetic Resonance ImagingEssen, Germany

**Keywords:** decisions under risk, executive functions, structural equation model, Game of Dice Task, self-control, monitoring

## Abstract

Executive functioning is supposed to have an important role in decision making under risk. Several studies reported that more advantageous decision-making behavior was accompanied by better performance in tests of executive functioning and that the decision-making process was accompanied by activations in prefrontal and subcortical brain regions associated with executive functioning. However, to what extent different components of executive functions contribute to decision making is still unclear. We tested direct and indirect effects of three executive functions on decision-making performance in a laboratory gambling task, the Game of Dice Task (GDT). Using Brand's model of decisions under risk (2006) we tested seven structural equation models with three latent variables that represent executive functions supposed to be involved in decision making. The latent variables were general control (represented by the general ability to exert attentional and behavioral self-control that is in accordance with task goals despite interfering information), concept formation (represented by categorization, rule detection, and set maintenance), and monitoring (represented by supervision of cognition and behavior). The seven models indicated that only the latent dimension general control had a direct effect on decision making under risk. Concept formation and monitoring only contributed in terms of indirect effects, when mediated by general control. Thus, several components of executive functioning seem to be involved in decision making under risk. However, general control functions seem to have a key role. They may be important for implementing the calculative and cognitively controlled processes involved in advantageous decision making under risk.

## Introduction

Many decisions in life are made under risk conditions. Under risk conditions, the available options potentially lead to suboptimal or negative consequence. It is often differentiated between two types of risk. Under ambiguous risk, the rules for positive and negative outcomes from different options are not explicitly provided to the decision maker. Here we concentrate on objective risk, which means that explicit information about the rules for the potential positive and negative consequences are available to the decision maker (see e.g., Yates and Stone, 1992; Bach and Dolan, 2012; Volz and Gigerenzer, 2012). Furthermore, probabilities for the occurrence of outcomes are explicit or can be calculated. An example is the decision of whether or not to take out an occupational disability insurance while knowing the probability to become unemployable as a consequence of an accident. Another example is the decision between either betting that one specific number will be thrown with a die (e.g., the “6”) or betting that one of two numbers will be thrown (e.g., the “6” or the “5”). When a person is confronted with a decision of this kind and the decision situation is new to him/her (i.e., non-routine), it should be advantageous for him/her to exert cognitive control before making the decision. Cognitive control should be useful for example for calculating probabilities before making the decision or—if several decisions have to be made—for developing a strategy and control behavior accordingly when making the choices. For exerting such cognitive control over behavior, executive functions are thought to be responsible (Norman and Shallice, 1986; Shallice and Burgess, 1993; Brand et al., 2006). Executive functioning subsumes a number of component processes associated with different prefrontal and subcortical brain regions, in particular those involved in fronto-striatal loops (see e.g., Andres, 2003; Jurado and Rosselli, 2007). While several neuropsychological studies supported the assumption that executive functions are involved in decision making under risk (e.g., Brand et al., 2005; Delazer et al., 2007; Euteneuer et al., 2009), the current study aims at investigating the impact of different subcomponents of executive functioning on decision-making performance.

**Figure 1:**
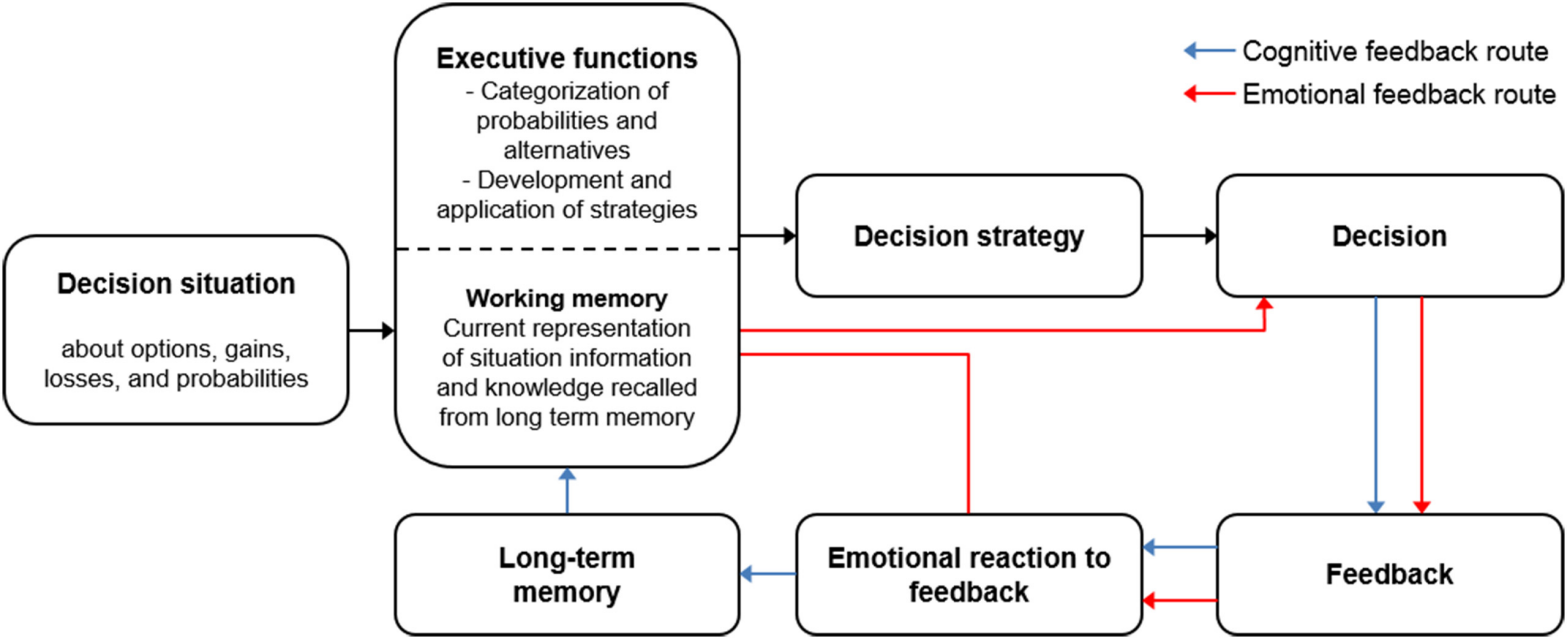
**The model of decision making under risk**. A major role of executive functions is suggested for the development and application of decision-making strategies as well as for processing of feedback. Blue arrows indicate the cognitive feedback route, red arrows the emotional feedback route. Adapted according to Brand et al. (2006).

One certain model (Brand et al., 2006) suggests that executive functions are involved in assessing probabilities, planning, and applying decision-making strategies, and using feedback to monitor strategies and to revise them if necessary. The main assumptions of the model are depicted in Figure 1.

The model suggests that executive functions process available information about the decision situation. Executive functions are furthermore involved in categorizing options, for example with respect to their potential gains and losses, as well as with respect to probabilities. Then executive functions guide the selection of information, the development of decision-making strategies, as well as the systematic application of the strategies. The model assumes that feedback about decisions' consequences can be processed on two routes: a cognitive and an emotional route. On the cognitive route (indicated by the blue arrows) executive functions are important for making use of the feedback. This involves consciously monitoring the decision strategy's consequence and revising the current strategy if necessary. On the emotional route (indicated by the red arrows) feedback can result in the development of automatic bodily signals (somatic markers, as suggested by Damasio, 1994, 1996; Bechara et al., 1997), which are experienced as hunches and guesses.

The relationship between executive functions and making decisions under objective risk conditions has frequently been investigated with the Game of Dice Task (GDT; Brand et al., 2005). The GDT is a measure of decision making under risk with explicit and stable rules for gains, losses, and their probabilities. In this computerized game of dice, participants have the goal to increase their fictitious starting capital of €1000 in 18 throws of a single die. Before each throw they have to guess which number will be thrown. The participants always have to decide whether they bet on one single number (possible gain/loss: €1000) or on combinations of two (€500), three (€200), or four (€100) numbers. If the number or one of the numbers of the chosen combination is actually thrown, the participants win fictitious money, if one of the other numbers is thrown, they lose money. As can be calculated, betting on one single number and betting on two numbers is highly risky, leading to high losses in the long run because winning probabilities are below 34%. Betting on three numbers or four numbers can be called lowly risky, because winning probabilities are at least 50%, in the long run leading to low gains/losses or at least retaining the starting capital. Good performance in the GDT has been shown to be affected by the ability to categorize and calculate probability information (e.g., probabilities and expected values; see Schiebener et al., 2011), the ability to develop and apply long term strategies based on these calculations (see Brand et al., 2008), and the ability to process the feedback about the decisions' consequences (particularly for monitoring the current strategy's success, and for revising it if necessary; see Brand et al., 2006, 2009a,b).

The GDT has been called one of the most important decision-making tasks (Gleichgerrcht et al., 2010). Decision-making performance in the GDT is reduced in patients with neurological diseases or psychiatric disorders (Svaldi et al., 2010; Bayard et al., 2011, 2010; Fond et al., 2013), in patients with executive dysfunctions, and in patients with prefrontal cortex damage (Brand et al., 2004, 2009b, 2005; Delazer et al., 2007; Euteneuer et al., 2009; Rossi et al., 2010). The role of deficits in executive functioning for decisions under objective risk was also supported in studies using other decision tasks than the GDT (Rogers et al., 1999; Manes et al., 2002; Sinz et al., 2008). Furthermore, relationships between executive domain tests and decision tasks were also found in brain-healthy participants (Brand et al., 2009a; Brand and Markowitsch, 2010; Del Missier et al., 2010, 2012; Gansler et al., 2011; Schiebener et al., 2011, 2012).

Underlining the important role of executive functions when making decisions under objective risk, the inferior and dorsolateral parts of the prefrontal cortex have been found to be activated during these decisions, as well as the anterior cingulate cortex and the posterior parietal cortex (Rogers et al., 1999; Labudda et al., 2008). Activations of the dorsolateral prefrontal cortex point toward roles of monitoring, categorization, and shifting (Burgess et al., 2000; Shafritz et al., 2005; Lie et al., 2006). The activation of the anterior cingulate cortex has been interpreted as a sign for conflict resolution processes during decision making (Labudda et al., 2008).

In summary, behavioral, and brain imaging studies point toward the important role of executive functions for decision making under objective risk, as measured for example by the GDT. However, little is known about *which* executive functions are crucial in determining this decision-making performance or how they may determine decision making in *interaction*. Thus, in the current study, we used a structural equation model (SEM) approach to investigate the role of different executive functions in predicting decision making in the GDT.

In the following, we explain the theoretical derivation for the SEM. For this, the main question is which components of executive functioning may particularly be involved in decision making under risk conditions. So far, researchers neither have agreed on a common definition of executive functions, nor have they agreed on a model or a theory defining how executive functions operate or which subcomponents of executive functioning exist. Nevertheless, the different existing definitions may be summarized as follows: Executive functions are a higher level cognitive system controlling and piloting behavior and cognition. They allow humans to regulate behavior and cognition in a planned, goal oriented, flexible, and effective way (Shallice and Burgess, 1996; Lezak et al., 2004; Jurado and Rosselli, 2007; Anderson et al., 2008).

For the current study's design we referred to a relatively frequently cited model of executive functioning that was suggested by Norman and Shallice (Norman and Shallice, 1986; Shallice, 1988). The model postulates that two modes are responsible for directing behavior and cognition: the contention scheduling (CS) mode and the supervisory attentional system (SAS). The CS mode is responsible for selecting routine schemas to perform cognitive and behavioral routine tasks (e.g., buying a coffee in the coffee bar every morning). A schema is a predefined set of operation units (e.g., standard motor operations, such as walking to the coffee bar, pulling out the wallet etc.), required for realizing the goal. The phenomenological materialization of a schema is a strategy (Stuss et al., 1995; Shallice, 2002). When a situation is new and no routine schemas are available or the routine schemas are inappropriate, the SAS needs to take control. It inhibits routine schemas and directs cognitive resources to find solutions for the given problem by developing appropriate new schemas. Suchlike executive control exerted by the SAS is suggested to be comprised of several component processes, that is different executive functions (Shallice and Burgess, 1996). Stuss et al. (1995) named five functions but pointed out that this list is not complete: “energization of schemata, inhibition of schemata, adjustment of contention scheduling, monitoring of schema activity, and control of ‘if-then’ logical processes” (p. 193). Examples for further components of executive functioning that have been mentioned in the literature are inhibition, shifting, updating, task management, monitoring, planning, coding, problem solving, or generating strategies (Borkowsky and Burke, 1996; Smith, 1999; Miyake et al., 2000; Elliott, 2003; Salthouse et al., 2003; Friedman et al., 2006, 2008; Jurado and Rosselli, 2007; Miyake and Friedman, 2012). To the best of our knowledge the CS-SAS model by Norman and Shallice (1986) is well accepted and is the only model which in detail describes the potential functioning of different components of executive functioning as well as the potential interactions between them. Therefore, we refer to this model for the theoretical derivation of the SEM. We use the general assumptions of the CS-SAS model (Norman and Shallice, 1986) and the model of decision making under risk (Brand et al., 2006) for inferring on a set of executive components which might potentially affect decision making under risk. Please note that—although based on theory—the choice of functions we investigate inevitably remains arbitrary to some extent. In other words, one might also find reasons to concentrate on investigating the differential roles of other components of executive functioning.

The model of decision making under objective risk (Brand et al., 2006) assumes that executive functions are important for processing information about the decision situation (categorizing alternatives according to gains, losses, and probabilities, understanding rules for gains and losses, etc.) and for developing and applying decision-making strategies. Based on these main assumptions we decided to investigate three executive functions, which may be involved in these processes:

1. G*eneral control*. General control stands for the ability to allocate attention according to a task's rules and goals. Thus, general control also involves the ability to inhibit the initiation of automatically imposing responses which are not in accordance with the task's rules and goals. One may also call this function attentional and behavioral self-control. In terms of the CS-SAS model general control is the ability to control whether a schema is activated and to shift to another schema if the imposing schema is inappropriate. In a novel decision situation under risk, for example when being confronted with the GDT for the first time, general control may be responsible for inhibiting the activation of a schema such as making generally unplanned choices or following an impulse of choosing the option were the highest monetary gain is offered. Furthermore, the model suggests that general control enables a shift to developing or acting out a new/revised strategy, if the current strategy appears inappropriate.
2. *Concept formation*. Concept formation subsumes the ability to form concepts of a non-routine task situation and manage solving of the task according to this concept. This ability may involve several processes, particularly categorization of information, detection of task rules, and maintenance of task solving strategies (often called set maintenance). In a decision situation under risk that is novel to the decider, this function may be involved in categorizing options with regard to potential gains and losses as well as with regard to probabilities (Brand et al., 2006). Furthermore, this function may be involved in using feedback in order to check and revise decision-making strategies (Brand et al., 2009a; Schiebener et al., 2011), and—when a strategy has been set up—this function may be involved in maintenance of behavior that is in accordance with the strategy (Brand et al., 2008, 2006).
3. *Monitoring*. We use the term monitoring for the ability to supervise whether one's own behavior is in accordance with and leads to a goal. The main ingredient of successful monitoring should be keeping information up-to-date in short-term memory. For example, when performing on one schema in a series of scheduled schemas, the schedule should need to be kept up-to-date. In terms of the CS-SAS model monitoring may be described as an online process of keeping in mind a superior goal while currently another schema is active. For example, in decision making under risk, a schema (i.e., a current decision-making strategy) may be active. The schema may for example be: Making a series of five decisions for the four number alternative in the GDT. In this example, monitoring may be important for keeping track of the current position in the action units of this schema (such as knowing that this is the third of five decisions for four-number combinations). At the same time it should be advantageous to keep in mind the task goal (reaching the best possible outcome) and to check the fit between the current strategy and the goal (e.g., by keeping in mind the consequences of previous success with the current strategy).

The current study has two aims. First, to examine the extent of impact each of these three components of executive functioning has on decision making under risk. Second, to examine whether the three components affect decision making under risk in interaction. Shallice and Stuss (2008) pointed out that some components of the SAS interact and that the SAS-component “attentiveness” (denoting the general allocation of processing resources) is responsible for implementing the cognitive or behavioral output. We regard attentiveness as being represented best by general control, because general control stands for the ability to allocate attention and to implement the behavioral output to be in accordance with a task's rules and goals. Thus, our behavioral output (decision making) may particularly be affected by general control. Monitoring (e.g., keeping task goals in mind) and concept formation (e.g., understanding and applying the task rules, categorizing stimuli, etc.) may be preconditions for being able to exert this general control in a way that it serves a task goal. Therefore, the effects of concept formation and monitoring on decision making may be mediated by general control abilities. For considering this hypothesis as being supported, we require the model fit of this mediation model to be significantly better than the fit of six other models assuming all possible predictor-mediator combinations predicting decision making under risk (see **Table 3**).

## Materials and methods

### Participants

We recruited 152 brain-healthy participants (66 males, 86 females), aged 18–75 years (*M* = 38.67 years, *SD* = 16.42 years) by local advertisement. Only 25 participants (16%) were students. They received €20 or course credits. Testing took place at the department of General Psychology: Cognition, at the University of Duisburg-Essen. Screening interview and self-report questionnaires were used to exclude participants with a history of neurological or psychiatric diseases. For participants over 50 the DemTect (Kalbe et al., 2004) was used to control for dementia (all scores were 13 or higher, indicating no signs of dementia). The study was approved by the local ethics committee of the Department of Computer Science and Applied Cognitive Science of the University of Duisburg-Essen. The committee decides on the ethical soundness of studies by referring to the declaration of Helsinki and the recommendation on ethics in psychological research published by the German Psychological Association. All participants gave their informed consent prior to the investigation.

### Instruments/procedure

All participants completed the GDT (Brand et al., 2005) and then a series of neuropsychological tests, involving executive functioning tests and the LPS subtest reasoning to estimate general intelligence (Horn, 1983). The tests of executive functions were chosen because they are face valid for covering one of the three functions each, as will be explained below for each task. Please note two things: First, there is no empirical data showing that the tasks cover these domains so far. Second, the tasks do not necessarily assess these functions exclusively, as described below. This is a typical problem of executive functioning tasks and is known as the task impurity problem (Burgess, 1997; Phillips, 1997). Thus, we can only argue—based on the task's structures—that the main variance in these tasks is produced by individual differences in the three functions (i.e., either general control, concept formation, or monitoring).

### Decision making under risk—game of dice task

In the computerized Game of Dice Task (Brand et al., 2005) the goal is to increase the fictitious starting capital of €1000 during 18 throws of one virtual die. Before each throw, participants guess which number (1–6) will be thrown. The die is thrown and the participants win if the guess was correct and lose if it was wrong. The bet can be placed on one single number or a combination of two, three, or four numbers. If the chosen number or one of the numbers among the chosen combination is thrown, participants win, if one of the other numbers is thrown, they lose. For example, the bet may be placed on two numbers, such as the “1” and the “2.” The die is thrown and €500 is won in case the number “1” or “2” is thrown. In case one of the other numbers is thrown (the “3,” “4,” “5,” or “6”), €500 is lost. Analogously, the participants can also bet on one, three, or on four numbers. The gains, losses (stable and permanently shown on screen), and probabilities (stable, not shown on screen) are:

- One single number: €1000 gain/loss (winning probability 1:6).
- Two numbers: €500 gain/loss (winning probability 2:6).
- Three numbers: €200 gain/loss (winning probability 3:6).
- Four numbers: €100 gain/loss (winning probability 4:6).

Participants are informed about all rules, the number of trials, and that they can continue playing even if their balance is negative. Gains and losses are accompanied by distinct sounds, and are presented on screen after each throw. The current balance and the number of played and remaining rounds are permanently kept up-to-date and shown on screen (Brand et al., 2005).

According to the convention, the options can be grouped into “high risk,” “disadvantageous” (one or two numbers with a winning probability less than 34%) and “low risk,” “advantageous” (three or four numbers with a winning probability of 50% and higher). Choosing the low risk options most probably leads to a positive overall capital in the long run, because winning probabilities are 50% or higher, promising at least to retain the starting capital of €1000. Choosing high risk alternatives normally results in a negative overall capital, because the winning probabilities are below 34%.

#### Main measures

As measure of GDT performance, a net score is used (the number of decisions for low risk options minus the number of decisions for high risk options). The net score is positive when more advantageous than disadvantageous decisions are made.

### General control

#### Color word interference test (CWIT)—interference trial

The interference trial of the CWIT (Stroop, 1935; Bäumler, 1985) is a paper task measuring attentional control, also called interference control. Participants are presented with a piece of paper showing a list of 72 color words. The meaning of each word differs from its ink color (color words and ink colors: red, blue, green, and yellow). Participants are asked to speak out the ink colors of the words one after the other as quickly and as accurately as possible.

In this task, good performance requires strong control over attention involving inhibition of the impulse to read out the word. Besides inhibiting the impulse it is necessary to shift to categorizing and naming the ink color of each word.

#### Main measure

We measured CWIT performance by the time the participants needed to complete naming the colors on the list (shorter times indicate better performance). We used the raw time instead of a residual or difference score which would subtract processing speed (as measured by reading- and color-trials of the CWIT). Given that we aimed to measure control over attentional processing resources, which also involves speed, we also allowed our measurement to involve processing speed.

#### Trail making test part B (TMT B)

The TMT B (Reitan, 1958; Reitan and Wolfson, 1995) assesses psychomotor control and loads particularly on inhibitory control and shifting. Participants are presented with a paper-sheet with encircled numbers (1–13) and letters (A–L). The task is to connect numbers and letters alternating in numerical, respectively alphabetical order, starting with 1, proceeding to A, followed by 2, B, etc., and ending with 13.

Comparable to the CWIT this task loads on attention control and requires inhibiting the automatic impulse of drawing the line to the next number or letter in the order. Instead, it is necessary to shift to another response category before drawing each new line.

#### Main measure

The TMT B performance is measured by the time needed to complete all connections (shorter times indicate better performance). Again, for the same reasons as with the CWIT, we used the raw time instead of a residual or difference score.

### Concept formation

#### Modified card sorting test (MCST)

The MCST (Nelson, 1976) is a modification of the Wisconsin Card Sorting Test (WCST; Berg, 1948). In the computerized task, the participants need to categorize options, detect rules and maintain sets. Participants are presented with a deck of cards. Each card shows a certain number (1–4) of shapes (square, circle, triangle, or star) in a color (blue, red, green, or yellow). The participants have to sort every appearing card to one of four target cards. However, they do not know what sorting rule to apply (sorting rules: by number of shapes, by shape, by color of shapes). Feedback (visual and acoustic) is given and can be used to find the correct sorting rule. After six cards are consecutively sorted correctly, participants are informed that the rule has changed. They then have to find out the new rule by trial and error.

The MCST has previously also been found to load on set shifting/cognitive flexibility and inhibition (see e.g., Miyake et al., 2000). Nevertheless, given the concept of the MCST, we assume that the three main abilities are categorization, rule detection, and set maintenance because participants have to use feedback to recognize a sorting rule for cards and have to apply the sorting rule according to different categories of card symbols. Without being able to categorize information, detect rules and maintain sets, it should be impossible to solve the task successfully. Inhibition and shifting may be involved but are probably not the main components.

#### Main measure

We used two measures: The number of perseverative errors (card is sorted according to the sorting rule of the previous completed category) and the number of non-perseverative errors (number of incorrectly sorted cards minus number of non-perseverative errors).

### Monitoring

#### Balanced Switching Task (BST)

The BST was developed to assess monitoring. The BST is a voluntary task switching paradigm similar to a task used by Arrington and Logan (2004). Furthermore, it shares some conceptual features with the Hotel Task (Manly et al., 2002), which is said to measure monitoring. In the Hotel Task different tasks have to be completed and participants have to switch between them in order to complete as much of each task as possible and to complete each of the tasks to comparable amounts. The Hotel task was also considered as a measure of monitoring for the current study, but was not chosen because of its complexity with regard to the different demands the subtask place on executive functions. We tried to keep the demands and difficulty of the subtasks in the BST as comparable as possible.

In the BST participants have four tasks. They can switch between them voluntarily. The explicit aim is to work on each of the four tasks to equal amounts. There are two sets of stimuli (set A: two-digit numbers from 01 to 99 and set B: abstract geometric shapes with diagonal hedging). Within the sets, the participants can work on one of two tasks at the same time. In set A, task 1 is to indicate whether the presented number is odd (press “d” on the keyboard) or even (press “f”). Task 2 is to indicate whether the number is below 50 (“j”) or above 50 (“k”). In set B, task 1 is to indicate whether the diagonal hedging within the shape is directed to the upper left (“d”) or to the upper right corner (“f”). Task 2 is to indicate whether the shape is oriented vertically (higher than broad, “j”) or horizontally (broader than high, “k”). With the space bar the participants can switch between the sets A and B. Within the sets, they can switch between the tasks 1 and 2, by switching between the response keys (“d,” “f”/“j,” “k”). Only one stimulus is presented at the same time. With each presented stimulus the participants have to apply only one of the four tasks.

The participants are informed that they have three aims:

- working on all tasks as equally often as possible.
- classifying the stimuli as correctly as possible.
- working on as many stimuli as possible (by making quick responses).

They are also informed that switching between the sets with the space bar results in a loss of time. This rule was used to increase the load on monitoring. One can assume that the rule motivates staying with one task for a longer time. Staying longer in one set should increase the cognitive effort of keeping in mind how long and how often they have worked on the other tasks before and the effort of remembering that further switches need to be made.

The duration of the task (two blocks of 4 min) and the stimulus presentation times (until response is made, but maximally 1000 ms) are not known by the participants. The inter-stimulus interval is 500 ms, a switch between sets A and B costs 1250 ms of the overall time. All subtasks as well as the overall task are practiced. For each participant the experimenters made sure during the practice trials that the task was fully understood.

After each of the 4-min blocks, feedback on participants' performance is provided with respect to the three aims. After the first block the participants are reminded of the four existing tasks and the assignment of the response keys.

We assume that performing well in the BST requires particularly monitoring abilities. Although other executive process such as shifting and inhibition may be involved, the main component that should be required to be able to distribute work on the four tasks to equal shares should be monitoring, because it has to be monitored continuously that there are other tasks to work on. Furthermore, participants have to monitor how often and how extensively they worked on the current and on the other tasks.

#### Main measure

We used a so called *deviation score*. This was computed for each of the two blocks (BST 1, BST 2). The deviation score formula was derived from the statistical formula for computing the standard deviation of a sample. The deviation score indicates the deviation from the optimally equal/balanced performance (0% deviation: optimal performance; 43% deviation: worst performance, i.e., working on only one of the tasks). For the score, the percentages of stimuli presentations within each of the four tasks were used as basis (e.g., number of stimuli presented in task 1 divided by the number of stimuli presented overall). In the formula below, this value is denoted by the variables taskA1, taskA2, taskB1, and taskB2. From this value the optimal value of equal performance (25% in each task) was subtracted and the result was squared. This was done for each task. The results were summed and then divided by four. From this result the square root was taken.

$$\begin{array}{l} deviation\;score=\surd \{[{\left(\text{taskA1}-25\right)}^{2}+{\left(\text{taskA2}-25\right)}^{2}\\ \;+{\left(\text{taskB1}-25\right)}^{2}+{\left(\text{taskB2}-25\right)}^{2}]/4\} \end{array}$$

### Statistical analyses

Statistical standard procedures were carried out with IBM SPSS Statistics (version 21.0, 2012, SPSS inc. IBM, Chicago). Pearson correlations were calculated to test for zero-order relationships between two variables. The data were controlled for outliers with four methods and with respect to all possible pairs of manifest variables: regression with a random variable, analysis of studentized *t*, SPSS case-wise methods, and visual control. Detailed information can be found in the Appendix. Given that no influential outliers were identified, the analyses were performed with all subjects.

The TMT B, MCST number of perseverative errors, and deviation from balance in BST block 1 and 2 were normalized with natural logarithmic transformation because of skewness above |1.00| or kurtosis above |3.00|.

The SEM analysis was computed with MPlus 6 (Muthén and Muthén, 2011). The maximum likelihood parameter estimation was applied. There were no missing data.

Model fits were evaluated by standard criteria (Hu and Bentler, 1995, 1999) for fit indices: χ^2^ test (significant values indicate that the data significantly deviate from the model), standardized root mean square residual (SRMR; values < 0.08 indicate a good fit between model and data), comparative fit indices (CFI/TLI; values > 0.90 indicate a good fit, values > 0.95 an excellent fit), and root mean square error of approximation (RMSEA; “test of close fit”; a value < 0.08 indicates acceptable fit). Furthermore, the model was compared to a baseline model. Significant χ^2^ values for the baseline model indicate that a theoretical model fits significantly better with the data than with the baseline model. For comparing models the χ^2^ model comparison test was used. Furthermore, for model comparison, the Bayesian Information Criterion (BIC) are reported for each model. Smaller BIC values indicate that a model explains the data better than a model with higher BIC values does (see e.g., Kaplan and Depaoli, 2012). For mediator analysis all variables included in the mediation were required to correlate with each other (Baron and Kenny, 1986).

## Results

The descriptive values of the sample's performances in the GDT and the executive tests are shown in Table 1.

**Table 1:** **Descriptive values of the sample's performances in estimated intelligence, GDT, and the executive functioning tests**.

	**Range**	***M***	***SD***	***Skewness***	***Kurtosis***
Intelligence^a^	85–140	114.98	11.42	−0.23	0.94
GDT net score^b^	−18–18	6.63	11.12	−0.83	−0.40
CWIT^c^	46–123	70.26	13.91	0.78	1.02
TMT B^c^^,^^d^	25–140	60.05	23.10	0.32	−0.32
MCST non-perseverative errors	0–27	8.60	6.06	0.89	0.12
MCST perseverative errors^d^	0–18	2.28	3.38	1.05	0.59
BST 1^d^^,^^e^	0.01–0.43	0.14	0.11	−0.012	−0.77
BST 2^d^^,^^e^	0.01–0.43	0.13	0.11	−0.015	−0.72

Abbreviations: GDT, Game of Dice Task; CWIT, Color Word Interference Test; TMT B, Trail Making Test part B; MCST, Modified Card Sorting Test; BST, Balanced Switching Task.

a Estimated with subtest reasoning of the Leistungsprüfsystem [German intelligence test battery].

b Net score (number of low risk decisions—number of high risk decisions).

c Time in seconds (higher scores represent worse performance).

d The variable was logarithmically transformed. For means and standard deviations we report the original values. The skewness and kurtosis values are those for the logarithmically transformed versions of the variables.

e Deviation score (percentage of deviation from optimally balanced performance on all four tasks).

**Figure 2:**
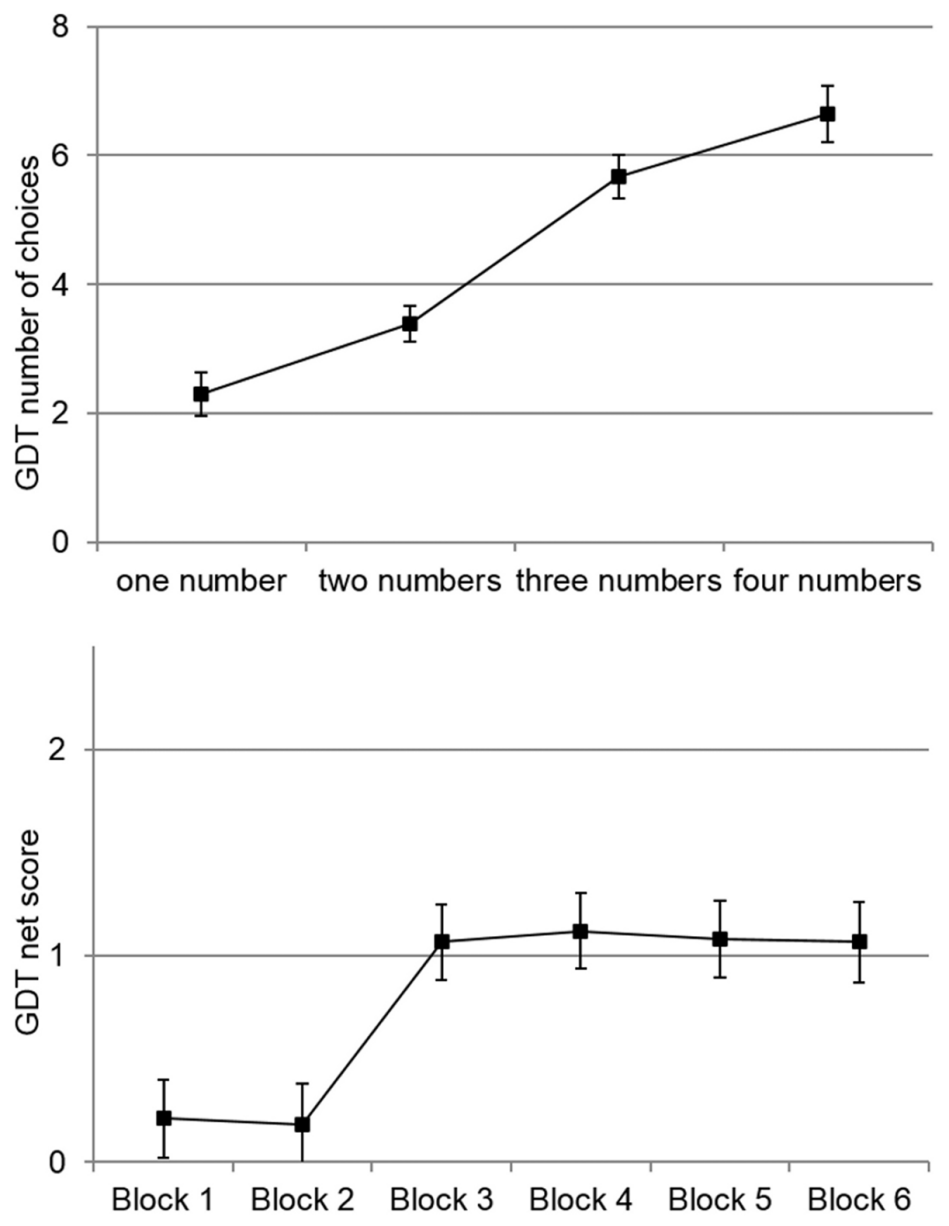
**Descriptive values of GDT behavior**. **Top**: Mean number of choices for the four risk alternatives. **Bottom**: Course of behavior over the task's duration with regard to net score (number of low risk choices—number of high risk choices) in six blocks á three decisions. Error bars are standard errors.

The GDT scores and the neuropsychological test scores are all in the normal range, comparable to other studies (Jensen and Rohwer, 1966; Lineweaver et al., 1999; Tombaugh, 2004; Sheridan et al., 2006; Verdejo-García and Pérez-García, 2007; Brand et al., 2009a). The average GDT net score around six indicates that the participants made on average about six more decisions for low risk options than for high risk options. However, there was high variance indicating that there were many participants deciding substantially more risky or substantially less risky. To get further insight into the general behavior in the GDT we analyzed the average frequency of choices for all four risk alternatives (one number, two, three, and four numbers) and the development of behavior over the task's course (separated into six blocks with three decisions each, as also done in Schiebener et al., 2013). See Figure 2 for the descriptive values.

As known from previous studies, there was a significant main effect of risk alternative, *F*_(2.47, 372.47)_ = 24.55, *p* < 0.001, partial η^2^ = 0.14, indicating that the participants made more choices with lower risk. Furthermore, there was a significant main effect of block, *F*_(3.94, 594.94)_ = 12.29, *p* < 0.001, partial η^2^ = 0.08. As observed by Schiebener et al. (2013) the participants made on average more risky decisions in the first two blocks and less risky decisions thereafter. The main correlations can be found in Table 2.

**Table 2:**
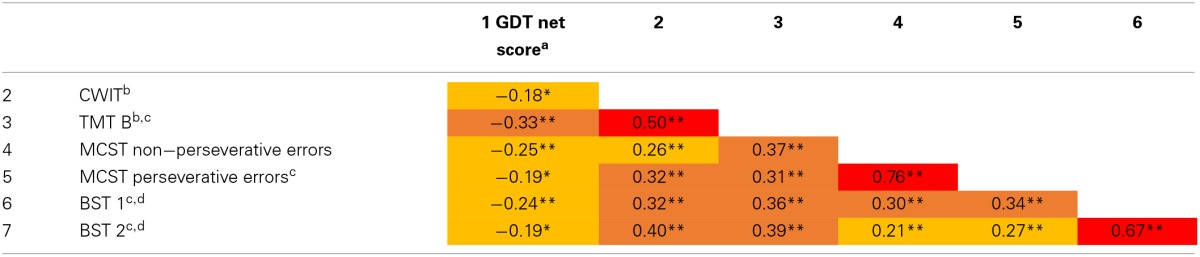
**Correlations between GDT net score and tests of executive functions**.

The color codes illustrate the effect sizes (see legend below).

Abbreviations: GDT, Game of Dice Task; CWIT, Color Word Interference Test; TMT B, Trail Making Test part B; MCST, Modified Card Sorting Test; BST, Balanced Switching Task.

^*^p = 0.05.

^**^p = 0.01.

Color code:

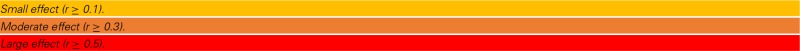

^a^Net score (number of low risk decisions—number of high risk decisions).

^b^Time in seconds (higher scores represent worse performance).

^c^The variable was logarithmically transformed.

^d^Deviation score (percentage of deviation from optimally balanced performance on all four tasks).

There were low to moderate significant correlations between the measures of executive functions and the GDT net score. Furthermore, there was an inverse correlation between age and the GDT net score (*r* = −0.20, *p* = 0.015), which is comparable to previous studies with the GDT (e.g., Brand and Markowitsch, 2010; Brand and Schiebener, 2013). Age was correlated with all scores of executive functioning (*r*s from 0.37 to 0.46, *p*s < 0.001). Within the CWIT the correlation between the time needed and the number of errors was not significant, *r* = 0.13, *p* = 0.111. (Note: We also calculated all correlations with Spearman's rho and Kendall's tau. None of the correlation coefficients substantially changed in comparison to the Pearson correlations. Furthermore, we controlled for age effects by adding age as an additional predictor in all SEMs. Including age, the fit indices of the models fell below the acceptability thresholds. Thus, age was not included in the following main analyses).

Before calculating the seven main models for addressing the two aims of the study, we verified the arrangement of the latent dimensions by testing nine control models. Please note that we did not aim at finding the one best possible model of all models that could have been created with the set or subsets of the variables involved. Instead, we tested nine alternative arrangements of the three latent dimensions to make sure that we do not find an arrangement of latent dimensions that fits significantly better than the three dimension model. Using χ^2^-model comparison, the nine were then compared to the “basic” three dimension model assuming no indirect effects (model 2). The nine models were:

- a model summing all manifest variables in one latent dimension.
- two models in which manifest variables were arbitrarily interchanged between the latent dimensions.
- three models consolidating the manifest variables of two dimensions.
- three models excluding one of the latent dimension.

In summary, the results were the following: Most of the models have insufficient model fit values and fit significantly worse with the data than did model 2. Only the models excluding one dimension fit comparably to model 2. Given that they did not fit better than model 2 and given that we aimed at comparing the impact of all three executive components on decision making, we stick with the three dimensions defined beforehand.

Table 3 shows the fit indices of the seven main models that were tested to address the two aims of this study.

**Table 3:**
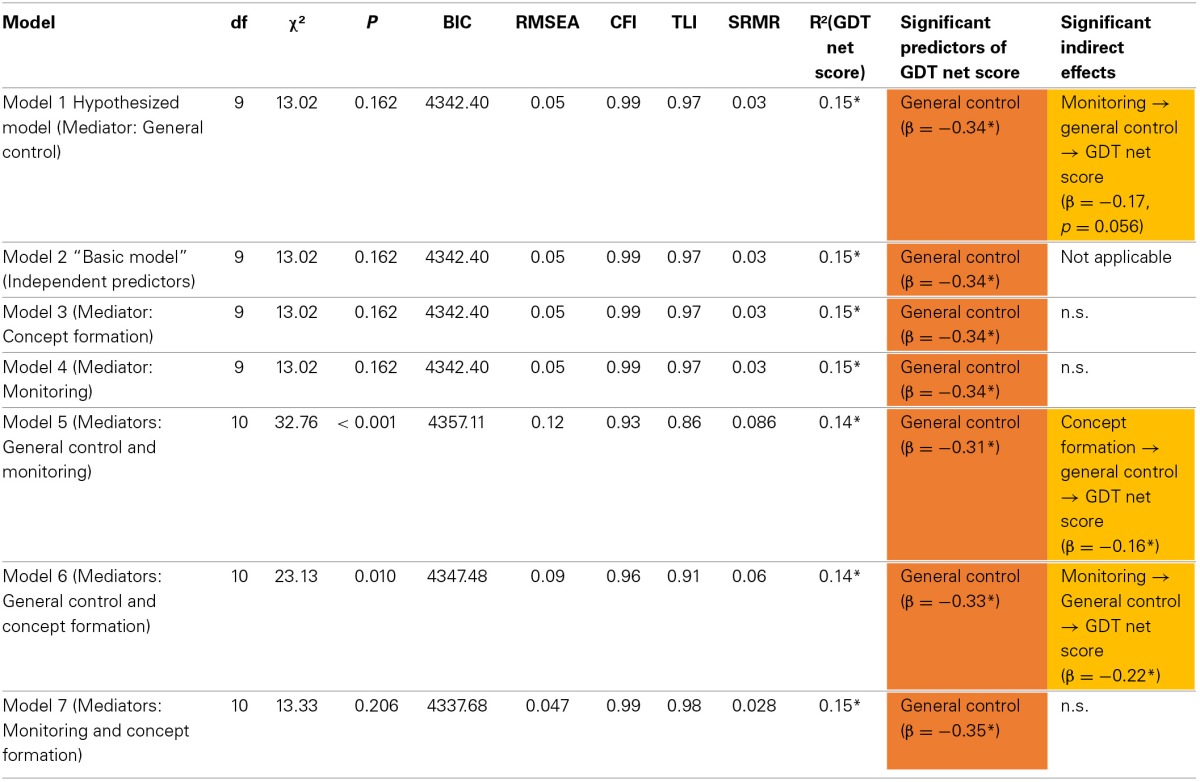
**The fit indices of the seven main SEMs**.

*p = 0.05.

Abbreviations: df, Degrees of freedom; BIC, Bayes Information Criterion; RMSEA, Root Mean Square Error of Approximation; CFI, Comparative Fit Index; TLI, Tucker Lewis Index; SRMR, Standardized Root Mean Square Residual; GDT, Game of Dice Task.

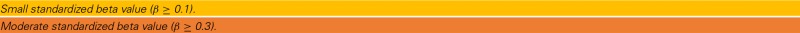

The models with only one mediator (model 1, 3, 4) as well as the model without a mediator (model 2) have equal model fits. This is no error. Instead, it indicates that none of these models can be preferred over the others. Only the two models assuming general control as mediator beside one of the other latent domains (model 5 and 6) fit significantly worse than the “basic model” (model 2) and the “hypothesized model” (model 1), smallest χ^2^_diff_ = 10.11, *p* < 0.001. Given that the hypothesized model (model 1) does not fit better than some of the other mediation models, there is no evidence for preferring the hypothesized model over the others.

In all models there is an obvious systematic pattern with regard to the role of the three executive domains. In all models general control is the main predictor of GDT performance. Furthermore, all significant indirect effects involve general control as the mediator.

These results can be summarized as follows. GDT net score can mainly be explained by general control. Concept formation and monitoring do not contribute to the variance in GDT net score unless they are mediated by general control.

## Discussion

This study aimed to investigate the extent of contribution of three different executive functions to the variance in decision-making performance under objective risk conditions, measured with the GDT. Furthermore, it aimed to examine the interactive influence of different executive functions on decision making under objective risk. Based on the model of decision making under risk (Brand et al., 2006) and the CS-SAS model of executive functioning (e.g., Norman and Shallice, 1986; Stuss et al., 1995; Shallice, 2002; Shallice and Stuss, 2008), we tested seven main SEM-models projecting different mediation assumptions with regard to the executive functions' effects on decision making under objective risk. Using different executive functioning tests, we tried to assess three executive functions: general control (supposed to load on general attentional and behavioral self-control), monitoring (supposed to load on supervision of behavior in accordance with task states and goals that are consequently kept in mind), and concept formation (supposed to load on categorization, rule detection, and set maintenance). At this point, we want to sensitize the reader once more to the fact that the assumption that the used executive tasks assess these three functions is based on no more than an evaluation of the tasks' face validities.

Generally, the amount of explained variance of decision-making performance (i.e., maximally 15%) supports the assumption that the ability to exert executive control over behavior and cognition is a predictor of decision-making performance. However, a large proportion of variance remains unexplained, indicating that there are further variables affecting decision-making performance.

The sub-domains of executive functions were all correlated with GDT performance on the bivariate level. Nevertheless, in the seven models performances in the tests used to measure monitoring and concept formation did no longer directly predict decision-making performance. Instead, the main direct predictor of decision making were individual differences in performances in the tests used to measure general control. Performances in tests used to assess monitoring and concept formation affected decision making only indirectly in model 1, 5, and 6, in which general control was defined as mediator. Thus, when assuming that the tests used are valid measures of the three executive domains, the results suggest that among different executive functions, general control has a key role for decision-making under risk. Concept formation and monitoring may also be involved but the two functions seem to be “unable” to affect decision-making performance without the involvement of general control.

These indirect effects indicate that components of executive functioning influence decision making in a mutually dependent relationship. A possible interpretation is that supervisory executive control processes are implemented via general control functions.

This interpretation is in line with the CS-SAS model (Norman and Shallice, 1986; Stuss et al., 1995). It generally states that executive functioning is required for developing a schema (e.g., a strategy) for the non-routine situations (e.g., new decision situations). In detail, supervisory functions, such as our monitoring and concept formation functions, are suggested to be responsible for development and application of schemas. Thereby they direct and schedule when a schema is implemented (e.g., a planned series of five decisions for four-number alternatives), other schemas have to be inhibited (e.g., the impulse to make decisions for alternatives offering higher potential gains), or new schemas are developed (e.g., a less risky strategy, when higher risks have previously led to negative consequences). Such control over schemas was suggested to be implemented by the general functions responsible for exerting executive control over cognitive processing resources (e.g., for inhibiting inappropriate schemas; Stuss et al., 1995).

The results fit well with the findings of an fMRI study by Labudda et al. (2008) in which participants had to make decisions between die bets similar to those in the GDT. During these decisions, activations of the dorsolateral prefrontal cortex have been interpreted as a correlate of the categorization of alternatives. Categorization processes were in our study operationalized with the MCST, which showed a marginally significant indirect effect on decision making in the GDT. The MCST is a modified version of the Wisconsin Card Sorting Test, during which the dorsolateral prefrontal cortex has been found to be active (Lie et al., 2006). Furthermore, the dorsolateral prefrontal cortex was active during one of the general control tasks, the CWIT (Kaufmann et al., 2005). Labudda and colleagues also reported that the anterior cingulate cortex was activated during decision making under risk. The anterior cingulate cortex has been associated with the allocation and maintenance of cognitive control (Holroyd and Yeung, 2012; Shenhav et al., 2013), and with conflict detection and resolution (Suchan et al., 2005). These functions are close to what we suggest to be measured by the general control tasks in the current study.

Future studies may further decompose the neurocognitive correlates of decision making under risk. Particularly, one may think of several other ways of investigating the role of different executive functions for decision making under objective risk. It may especially be considered a theoretical progress if studies succeed in delineating the differential roles of very basic executive functions for decision making (e.g., the roles of inhibition, updating, shifting, coding, etc.).

Beyond enhancing our understanding of the contribution of different executive functions to decision making, the observations in the current study can be interpreted with respect to the interplay of different executive functions during performance on more complex tasks. Many important studies have identified separate roles of different executive functions for domains of human performance, such as intelligence (Friedman et al., 2006), decision making (Del Missier et al., 2010; Gansler et al., 2011), or complex executive tasks (Miyake et al., 2000). The results of the current study extent the findings of these previous studies by pointing out that the effect of specific executive functions on a more complex task can be mediated by other executive functions.

### Conflict of interest statement

The authors declare that the research was conducted in the absence of any commercial or financial relationships that could be construed as a potential conflict of interest.

## Figures and Tables

**Figure A1:**
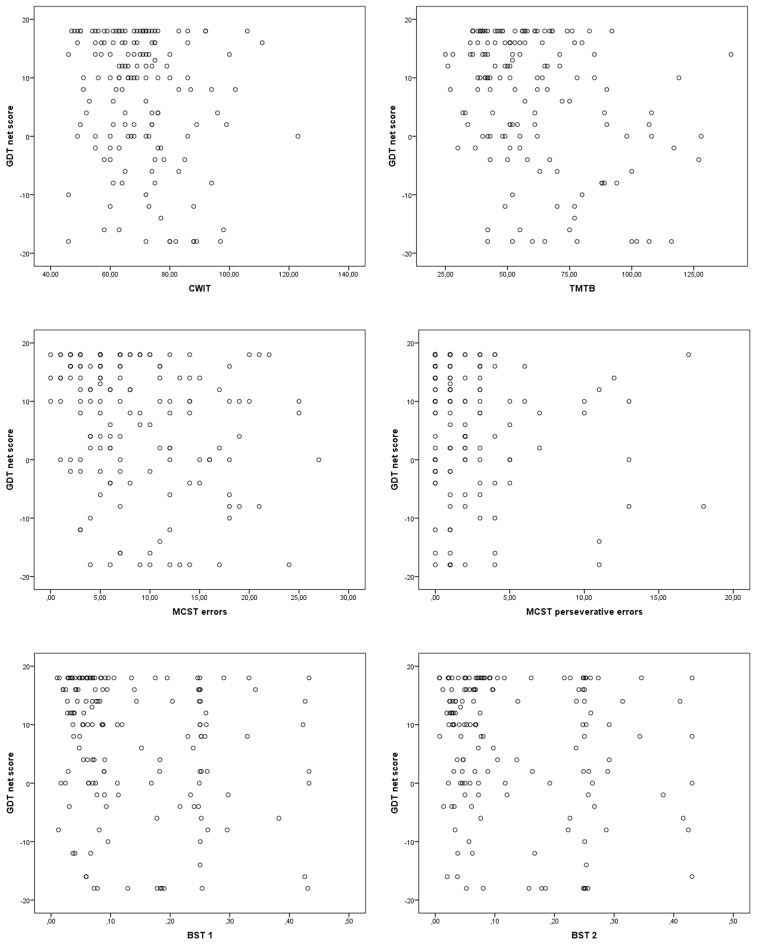
**The scatterplots between GDT net score and the scores of the executive functioning tests**.

## Appendix

Information on outlier detection methods (regression with a random variable, analysis of studentized *t*, SPSS case-wise methods, and visual control): In the regressions with the random variable, only the correlation with BST 2 was significant, *r* = 0.16, *p* = 0.049, but regarding the scatterplot between the random variable and BST 2 no outliers could be identified. All other correlations with the random variable were not significant, *r*s < 0.12, *p*s > 0.144. There were studentized *t*s beyond |3.00|, but in none of the cases their removal changed the correlation between the two variables remarkably. Thus, following the procedure also used by Miyake et al. (2000), these cases were not removed. SPSS case-wise methods revealed no cases extremely influencing the regressions between any pair of variables. An additional visual control revealed no outliers either. However, single cases at the edges of the distribution were tentatively removed. In none of the cases the correlation between the two variables was changed remarkably. The scatterplots including the dependent variable, GDT net score, can be found in Figure A1. All other scatterplots can be requested from the first author.
